# Pyrosequencing-Based Analysis of the Mucosal Microbiota in Healthy Individuals Reveals Ubiquitous Bacterial Groups and Micro-Heterogeneity

**DOI:** 10.1371/journal.pone.0025042

**Published:** 2011-09-22

**Authors:** Pei-Ying Hong, Jennifer A. Croix, Eugene Greenberg, H. Rex Gaskins, Roderick I. Mackie

**Affiliations:** 1 Department of Animal Sciences, University of Illinois, Urbana, Illinois, United States of America; 2 Department of Pathobiology, University of Illinois, Urbana, Illinois, United States of America; 3 Division of Nutritional Sciences, University of Illinois, Urbana, Illinois, United States of America; 4 Institute for Genomic Biology, University of Illinois, Urbana, Illinois, United States of America; 5 Institute for Digestive Health, Carle Foundation Hospital, Urbana, Illinois, United States of America; University of Hyderabad, India

## Abstract

This study used 16S rRNA-based pyrosequencing to examine the microbial community that is closely associated with the colonic mucosa of five healthy individuals. Spatial heterogeneity in microbiota was measured at right colon, left colon and rectum, and between biopsy duplicates spaced 1 cm apart. The data demonstrate that mucosal-associated microbiota is comprised of Firmicutes (50.9%±21.3%), Bacteroidetes (40.2%±23.8%) and Proteobacteria (8.6%±4.7%), and that interindividual differences were apparent. Among the genera, *Bacteroides, Leuconostoc* and *Weissella* were present at high abundance (4.6% to 41.2%) in more than 90% of the studied biopsy samples. *Lactococcus*, *Streptococcus*, *Acidovorax*, *Acinetobacter*, *Blautia*, *Faecalibacterium, Veillonella*, and several unclassified bacterial groups were also ubiquitously present at an abundance <7.0% of total microbial community. With the exception of one individual, the mucosal-associated microbiota was relatively homogeneous along the colon (average 61% Bray-Curtis similarity). However, micro-heterogeneity was observed in biopsy duplicates within defined colonic sites for three of the individuals. A weak but significant Mantel correlation of 0.13 was observed between the abundance of acidomucins and mucosal-associated microbiota (P-value  =  0.04), indicating that the localized biochemical differences may contribute in part to the micro-heterogeneity. This study provided a detailed insight to the baseline mucosal microbiota along the colon, and revealed the existence of micro-heterogeneity within defined colonic sites for certain individuals.

## Introduction

As an extension of the Human Genome Project, the Human Microbiome Project (HMP) was initiated to examine the microorganisms that human hosts harbor. One of the important objectives of HMP is to address the relationship between diseases with changes in the human microbiome [1]. The working hypothesis behind this objective is that host health status reflects the presence or abundance of certain microbial groups, and that these microbial groups can contribute either directly or indirectly to disease susceptibility [1]. To illustrate, numerous recent studies examined the gut microbiota present in various disease models: obesity [2], [3], [4], [5], and gastrointestinal diseases including ulcerative colitis and colorectal cancer [6], [7], [8]. Although promising results have been reported for some disease models, in particular obesity, the correlation between health status and mutualistic microbiota are still plagued by numerous confounding factors such as differences in host genetics, the dietary regimen, and environmental exposures. The approach of treating each person as their individualized and internalized control overcomes these potential confounding effects and technical challenges. This is particularly relevant in gastrointestinal disease models, which typically involve localized inflamed sites and adjacent healthy tissues. By comparatively examining mucosal-associated microbes present in both healthy and diseased sites within the same individual, the differences in the microbial communities can be better evaluated for its correlation to health status. However, prior to this, it is imperative to examine the baseline microbial diversity in each individual, and determine how the microbial community varies along the intestinal tract (i.e., longitudinal axis) and within sites of close proximities (i.e. small scale biogeographical differences).

Past studies have relied on molecular-based fingerprinting methods like denaturing gradient gel electrophoresis (DGGE) and terminal restriction fragment length polymorphism (T-RFLP) to examine the mucosal-associated microbiota [9], [10], [11]. It was shown that the predominant bacterial phyla in mucosa biopsy samples included Firmicutes, Bacteroidetes and Proteobacteria [11], and the microbial community remained relatively homogenous throughout the intestinal tract [9], [10]. However, fingerprinting methods are generally only able to identify predominant commensal microbiota (>1% of total microbial community), and this level of technical resolution means that DGGE and T-RFLP may not provide a representative evaluation of the microbial spatial distribution.

This study revisited the baseline diversity of the mucosal-associated microbiota by utilizing high-throughput sequencing technology (i.e., 16S rRNA-based pyrosequencing). A total of 165,953 sequences (approximately 4741±776 sequences per sample) were obtained, and evaluated for the microbial community that is associated with the colonic mucosa of five healthy individuals. Besides elucidating the baseline mucosal microbiota in these five individuals, the spatial heterogeneity in a longitudinal axis (i.e., right colon, left colon and rectum) and between biopsy duplicates in close proximity (i.e., spaced 1 cm apart) were also examined in the healthy colonic mucosa.

## Materials and Methods

### Ethics statement

All procedures were approved by the Carle Foundation Hospital (No. 0670) and University of Illinois (No. 07232) Institutional Review Boards. Subjects provided written informed consent prior to colonoscopy.

### Sample collection

Five healthy subjects (2 women, both of age 50, and 3 men, of ages 50, 73 and 74), undergoing routine screening colonoscopy at Carle Foundation Hospital (Urbana, IL USA), were recruited for this study. Two of these individuals (A and B) were spousal partners living in the same household. Individuals were prescribed 1.9 L of an oral polyethylene glycol (PEG) and electrolyte solution (Golytely; Braintree Laboratories, Braintree, MA) for bowel cleansing prior to colonoscopy. The standard prescription includes consuming 240 ml Golytely per time over the course of 8 h on the evening before colonoscopy. The recruited individuals also fast overnight (without breakfast), and had not been on antibiotics for at least 30 days prior to sample collection. After obtaining informed consent, subjects were given opioid analgesic (IV fentanyl) and amnesiac (midazolam), respectively, to lessen discomfort during the procedure. Four mucosa biopsies, approximately 1 cm apart, were collected from right colon (ascending colon), left colon (descending colon) and terminal colon (rectum), respectively, using Boston Scientific Radial Jaw™Jumbo forceps (3.2 mm). Of the four biopsies taken from each site, two adjacent biopsies of epithelial mucosa were immediately frozen in liquid nitrogen and used for microbial analysis via 454 pyrosequencing. The remaining two biopsies were fixed in Bouin's solution for mucin histochemistry analysis. Fixed biopsies were sent to the Carle Foundation Hospital Pathology Services Laboratory (Urbana, IL USA) for processing, embedding, and sectioning. In total, 30 biopsy samples were collected. Five pooled stool samples from a total of 20 other healthy individuals of the same age group were also collected to provide an outgroup comparison against the colonic microbial community. Subjects who submitted stool samples had been informed of the stool sampling procedure. Briefly, stools were self-collected at home by the enrolled individual, and were immediately immersed in 100% ethanol for storage at room temperature. The stools were then transported to laboratory within 24 h, and immediately frozen at −20°C for storage.

### Demographic information

Demographic information of the five subjects is summarized in Table 1. In brief, all subjects had no known history of gastrointestinal disease. However, subject C had mild diverticulosis and a 7 mm flat adenomatous polyp in the sigmoid colon. Subject E had diverticulosis in right colon and sigmoid colon. Endoscopic findings confirmed that all subjects were free of gastrointestinal diseases, and none of the mucosa tissues sampled exhibited abnormalities. To identify acidomucins (sialomucins and sulfomucins), biopsy sections were stained with high iron diamine (HID) and alcian blue (AB), pH 2.5, as previously described [12], and counterstained with nuclear fast red for 2 min. The stained biopsy sections were then examined with a Zeiss Axiovert 200M Microscope and the Mosaix module in Axiovision 4.5 software. The Automeasure module in Axiovision 4.5 was used to select and quantify the area of sialomucin and sulfomucin within goblet cells based on pixel color. The scoring system was executed as described previously [13]. In brief, the area of sialomucin and sulfomucin within goblet cells was measured, and then normalized to the area of epithelium containing the quantified mucin. A scoring index (0–3) was formulated to categorize the percentage of positive staining for sialomucin and sulfomucin, respectively (Table 1).

**Table 1:** Demographic information of the five individuals.

					Average score ± Standard Deviation					
					Sulfomucins			Sialomucins		
Subject	Age	Gender	Race	Endoscopic Findings	RC	LC	RE	RC	LC	RE
*A	50	F	Caucasian	Normal ileum, colon and rectum	2.2±0.3	2.3±0.8	2.1±0.7	1.3±0.6	1.1±0.2	2.4±0.5
*B	50	M	Caucasian	Normal ileum, colon and rectum	1.8±0.3	1.8±0.6	1.6±0.7	1.3±0.8	1.0±0.0	1.7±0.6
C	73	M	Caucasian	Mild diverticulosis and 7 mm flat adenomatous polyp in sigmoid colon. Normal ileum, colon and rectum.	1.9±0.2	1.7±0.6	1.3±0.5	1.5±0.4	2.4±0.8	2.7±0.6
D	50	F	Caucasian	Normal ileum, colon and rectum	1.8±0.8	1.8±0.5	1.3±0.5	2.1±1.0	1.0±0.0	1.3±0.3
E	74	M	Caucasian	Diverticulosis in right colon and sigmoid colon. Otherwise normal ileum, colon and rectum.	2.0±0.0	2.2±0.8	1.8±0.8	1.2±0.4	1.8±0.5	1.8±0.5

Mucin staining was done to determine the abundance of sulfomucins and sialomucins in the epithelium. The abundance of mucin stained goblet cells was further categorized according to a scoring index, where a score of 0 denotes no staining of that particular mucin type in epithelium, a score of 1 denotes 1–10% staining of that particular mucin type in epithelium, a score of 2 denotes 11–50% of staining of that particular mucin type in epithelium, and a score of 3 denotes >50% staining of that particular mucin type in epithelium. RC, LC and RE denote right colon, left colon and rectum, respectively.

*denotes spousal partners living in the same household.

### DNA extraction, barcoded PCR and 454 pyrosequencing

Genomic DNA was extracted using QIAamp DNA Stool Mini Kit (Qiagen, Valencia, CA). Modifications were made to the extraction protocol to enhance recovery of the gram-positive bacteria. Briefly, samples were added with enzymatic lysis buffer, lysozyme and achromopeptidase, and incubated at 37°C for 1 h. Proteinase K and lysis buffer AL were then added, and the suspension was incubated at 56°C for an additional 30 min. Genomic DNA was then subjected to spin column purification and elution. The concentration of extracted genomic DNA was measured with a Qubit fluorometer (Invitrogen, Carlsbad, CA). Samples for 454 pyrosequencing were amplified for the 16S rRNA hyper-variable regions (V4 to V5) with universal forward 519F (5′-Fusion A-Barcode -CAGCMGCCGCGGTAATWC-3′) and reverse 926R (5′-Fusion B-Barcode- CCGTCAATTCMTTTRAGTT-3′) primer pairs (www.roche.com). PCR reaction mixtures comprised 1 ng of genomic DNA, 25 µl of Premix F (Epicentre Biotechnologies, WI), 200 nM (each) of forward and reverse primers, 0.5 U of Ex *Taq* DNA polymerase (Takara Bio, Japan), and the volume added up to 50 µl with molecular-biology grade water. PCR with 35 cycles of thermal program (denaturation, 95°C for 30 s; annealing, 55°C for 45 s; and extension, 72°C for 60 s) was performed. The use of 35 thermal cycles was determined based on preliminary Q-PCR analysis, which denoted a threshold cycle ranging from 29–38 cycles was required to exponentially amplify the acetogenic, sulfate-reducing and methanogenic microbial populations from 1 ng of genomic DNA (data not shown). All amplicons were gel-excised, concentrated and purified with Wizard DNA purification kit (Promega, Madison, WI).

### Pyrotag handling and analysis

454 pyrosequencing was carried out on 454 FLX Titanium (Roche, Switzerland). The paired-end pyrosequencing services were provided by Roy J. Carver Biotechnology Center, University of Illinois. A total of 165,953 16S rRNA sequences (also referred as 16S pyrotags) were obtained and sorted based on their respective barcodes to form a total of 35 pyrotag libraries. Raw sequence reads were checked for their quality to minimize the effects of random sequencing errors. Briefly, quality check included the elimination of sequences that did not perfectly match the proximal PCR primer, and those with short sequencing length (<150 nt). All 16S pyrotags were then removed of their primers, barcodes, and adaptor sequences, and had an average read length of 369 nt after trimming (Table S1). 16S pyrotags identified with reverse orientation were also reverse complemented on RDP Pipeline Initial Process [14]. Processed pyrotags were then aligned based on RDP Infernal [14].

### Taxonomical classification and statistical analysis

RDP Classifier (version 10.21) was used for taxonomical assignments of the 16S pyrotags at 95% confidence level [14]. Primer-E worksheets that detailed the percentage abundances of individual bacterial genera were collated, and subsequently analyzed by multidimensional scaling (MDS) with Primer-E version 5 (http://www.primer-e.com/). Bray-Curtis dissimilarity matrix and mantel correlation analysis were calculated using the “vegdist” function in the Vegan package 1.17-3 in R (https://r-forge.r-project.org/projects/vegan/) on the genus-level community matrix. In addition, RDP Lib Compare was used to estimate the probability of observing differences in the abundance of a given phylogenetic taxon [15].

### Rarefaction curves and identification of shared OTUs

Aligned sequences for each sample were generated with their individual cluster files based on the RDP pyrosequencing pipeline. The cluster files were in turn used to generate rarefaction curves that defined the number of operational taxonomic units (OTUs, identified at 97% sequence similarity level) with respect to the total number of pyrotags read (Figure S1A). Regression analysis was also performed with Sigma Plot to fit the rarefaction curves into double rectangular hyperbola curve models (Table S2). Based on the regression curves, the number of OTUs identified based on 4000 pyrotags were noted for comparison of microbial richness (Figure S1B). A combined cluster file that includes all the mucosal-associated pyrotag libraries was also generated. Dereplication at 97% gene similarity level was performed to determine the unique OTUs present in all the mucosal-associated pyrotag libraries [14]. Given the relatively small number of individuals in our study (n = 5), a more stringent criteria was imposed when defining the ubiquitous bacterial groups. A Perl script was used to sort the OTUs that were shared by (i) >90% of the 30 biopsy samples, and (ii) were present in at least one of biopsy duplicates retrieved at a particular colonic site of the individual. The OTUs were blasted for their identity on RDP and then clustered into their respective bacterial groups.

## Results

### Interindividual differences in the abundance of mucosal-associated microbiota

**Figure 1:**
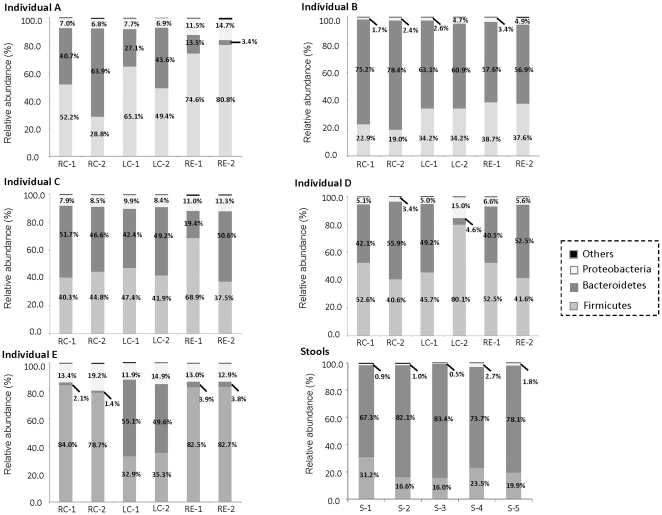
Bacterial phyla in colonic biopsy samples of five individuals and stool pool. Firmicutes, Bacteroidetes and Proteobacteria represent the three predominant phyla, and their respective abundance was listed accordingly. Y-axis denotes relative percentage abundance with respect to total Bacteria. Abbreviations RC-1, LC-1 and RE-1 denote biopsy duplicate 1 from right colon, left colon and rectum, respectively. Abbreviations RC-2, LC-2 and RE-2 denote biopsy duplicate 2 from right colon, left colon and rectum, respectively. Abbreviations S-1 to S-5 denotes the five pooled stool samples.

Both mucosal-associated and stool microbiota were comprised of three main phyla: Firmicutes, Bacteroidetes and Proteobacteria. All three phyla were consistently present, albeit at varying abundances in different individuals (Figure 1). Interindividual differences were apparent in the mucosal-associated microbiota (Bray-Curtis dissimilarity  =  0.50±0.11, Figure S2), and the proportion of Bacteroidetes with respect to Firmicutes varied among the individuals studied. For example, Firmicutes were present in individual A at abundances ranging from 28.8% to 80.8% of total microbial community, while the abundance of Bacteroidetes in the mucosa of this individual ranged from 3.4 to 63.9% of total microbial community. Individual E also harbored significantly higher proportions (>21-fold) of Firmicutes compared to Bacteroidetes in the right colon and rectal mucosa (Figure 1). In contrast, individual B had a higher abundance of Bacteroidetes (56.9 to 78.4% of total microbial community) in all of the sampled biopsies compared to the Firmicutes (19.0 to 38.7% of total microbial community). Besides Firmicutes and Bacteroidetes, the abundance of Proteobacteria (1.7% to 19.2% of total microbial community) also varied inter-individually, but was generally much lower in abundance compared to Firmicutes and Bacteroidetes (Figure 1). The three predominant phyla were mainly comprised of bacterial classes Bacilli (35.2%), Clostridia (14.4%), Bacteroidia (40.3%), Betaproteobacteria (2.3%) and Gammaproteobacteria (5.8%). In addition, unclassified Bacteria and the phylum Actinobacteria were also present in low abundance (<1% of total microbial community) in all of the examined biopsy samples. Rare phyla, namely Cyanobacteria, Fusobacteria, Acidobacteria, Verrucomicrobia, Chloroflexi, TM7, Nitrospira, Lentisphaerae, Synergistetes, Planctomycetes, Deinococcus-Thermus and Gemmatimonadetes, sporadically occurred at low abundance (approximately 0.1% of total microbial community) in some individuals (data not shown).

### Bacterial groups present in >90% of studied biopsy samples

Ubiquitous bacterial groups that were present in >90% of the 30 studied biopsy samples were further evaluated and identified based on the consensus model OTU sequences (Material S1). These bacterial groups were also present in at least one of the biopsy duplicates retrieved at a particular colonic site of an individual. The genus *Bacteroides* and lactic acid bacteria *Leuconostoc* and *Weissella* were present at high abundance in all individuals, ranging from 15.3% to 41.2%, 4.6% to 18.8%, and 6.4% to 24.1% of total microbial community, respectively (Table 2). Besides *Leuconostoc* and *Weissella*, other lactic acid bacteria including *Lactococcus* and *Streptococcus* were also present at an average abundance ranging from 0.23% to 6.4% of total microbial community. The bacterial genera that were ubiquitously present also consisted of the genera *Acidovorax*, *Acinetobacter*, *Blautia*, *Faecalibacterium*, *Veillonella*, and several unclassified bacterial groups, although these latter genera were present at relatively lower abundance than *Bacteroides* and the lactic acid bacteria (Table 2).

**Table 2:** Bacterial groups that were ubiquitously present in the mucosa of the five individuals.

Phylogenetic affiliations	Average percent abundance with respect to total microbial community ± standard deviation across the three sampled sites in the same individual				
	A	B	C	D	E
**Firmicutes**					
*Blautia*	0.16±0.05	0.16±0.05	0.50±0.16	0.53±0.29	0.27±0.24
*Faecalibacterium*	0.99±0.85	1.31±0.39	0.38±0.15	0.89±0.47	0.43±0.54
*Lactococcus*	5.55±2.33	1.40±0.77	3.37±1.71	3.24±2.98	6.35±3.83
*Leuconostoc*	15.61±10.11	4.56±2.23	10.33±5.03	9.98±8.62	18.79±10.24
*Streptococcus*	0.69±0.57	0.23±0.16	0.84±0.35	0.50±0.46	1.43±0.42
*Weissella*	20.64±13.48	6.35±3.35	13.67±6.50	13.32±12.11	24.12±13.16
*Veillonella*	0.41±0.38	0.14±0.12	0.38±0.11	0.32±0.33	0.57±0.32
Unclassified Clostridiales	3.76±2.57	4.42±0.83	6.96±0.95	6.95±4.25	2.85±2.56
**Bacteroidetes**					
*Bacteroides*	28.07±20.75	26.93±13.94	41.18±11.60	18.39±9.37	15.26±21.65
**Proteobacteria**					
*Acidovorax*	0.07±0.07	0.02±0.02	0.09±0.10	0.03±0.04	0.10±0.10
*Acinetobacter*	1.80±1.24	0.54±0.35	1.50±0.78	1.29±1.46	3.37±2.23
Unclassified Enterobacteriaceae	3.52±0.87	0.68±0.47	3.37±1.25	1.39±1.32	3.81±1.64
Unclassified Pasteurellaceae	0.31±0.33	0.61±0.23	2.19±0.94	0.07±0.05	1.26±1.61

Ubiquity was defined based on the presence of related OTUs in (i) >90% of the 30 biopsy samples, and (ii) were present in at least one of biopsy duplicates retrieved at a particular colonic site of the individual. The OTUs were blasted for their phylogenetic affiliations on RDP. The consensus model OTU sequences can be found in Material S1.

### Differences in mucosal-associated microbiota along the colon

**Figure 2:**
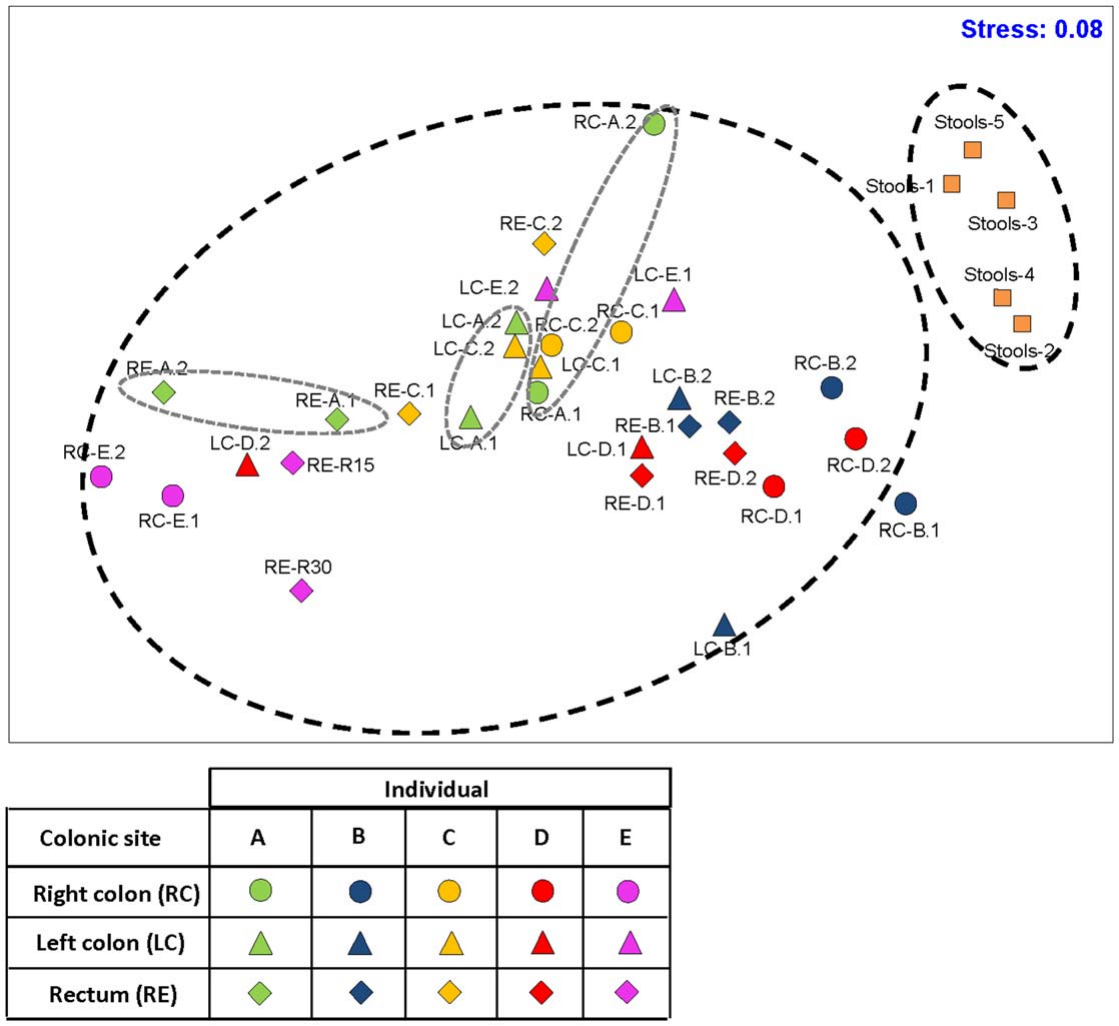
Multidimensional scaling plot (MDS) of bacterial lineages in the mucosal-associated and stool microbiota. Mucosal-associated microbiota within individuals varied along and within the sampling sites. The mucosal-associated microbiota is distinctly clustered apart from the stool microbiota.

**Figure 3:**
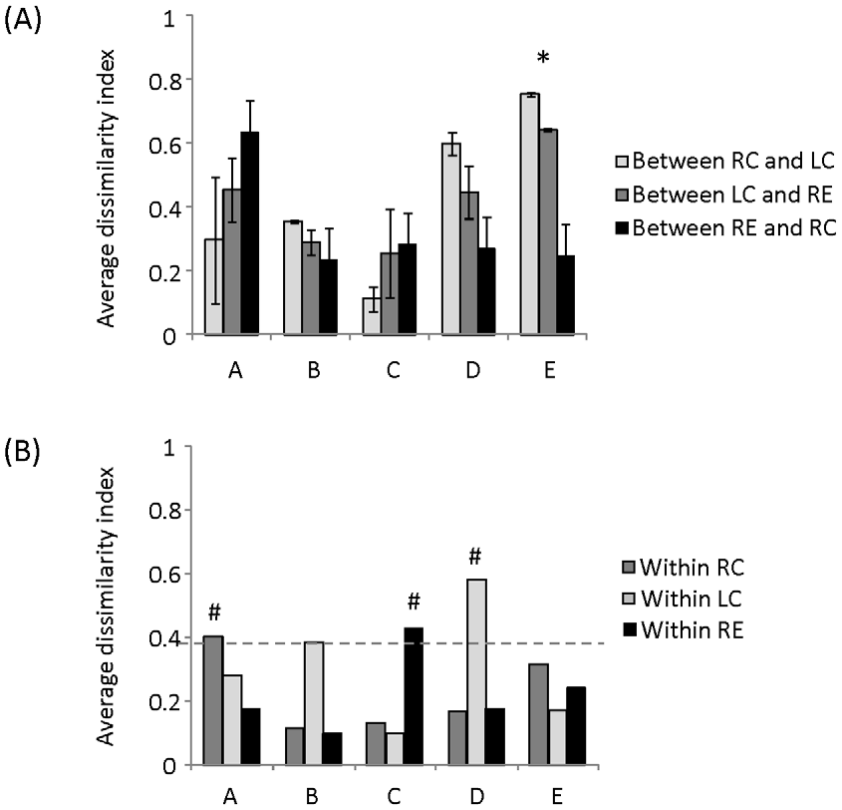
Bray-Curtis dissimilarity indices (A) along the GI tract, and (B) within biopsy duplicates of individuals A, B, C, D and E. Abbreviations RC, LC and RE denote right colon, left colon and rectum, respectively. * denotes that individual E exhibited significant heterogeneity in mucosal-associated microbiota in the three colonic sites. # denotes that biopsy duplicates at that particular colonic location was greater than the 0.39 Bray-Curtis dissimilarity index.

Distinct differences in the taxonomical profiles of mucosal-associated microbiota and pooled stool microbiota resulted in two groups that clustered apart on the multidimensional scaling plot (MDS) regardless of the host origins (Figure 2), suggesting that the mucosal-associated microbiota examined in this study were not contaminated with feces. Within an individual, the mucosal-associated microbiota in the rectum, left and right colon clustered separately in the MDS (Figure 2). Further evaluation of the Bray-Curtis dissimilarity index along the colon showed relative homogeneity in the mucosal-associated microbiota. On average, the Bray-Curtis dissimilarity index obtained for all individuals was 0.39±0.05 (i.e., 61% similarity) in mucosal-associated microbiota along the colon, although the extent of dissimilarity differed among individuals (Figure 3A). To illustrate, mucosal-associated microbiota of individuals B and C was on average 0.25±0.05 dissimilar (i.e., sharing 75% similarity) along the different sites of colon. In the three remaining individuals, the Bray-Curtis dissimilarities ranged from 0.44 to 0.55 (i.e., <56% similarity), indicating a relatively higher extent of heterogeneity in mucosal-associated microbiota along the colon for these three individuals. One-way ANOVA was carried out to determine if the Bray-Curtis dissimilarity among the sampling sites of each individual varied significantly. With the exception of individual E who harbored significantly higher heterogeneity in mucosal-associated microbiota along the colon (P-value  =  0.01), the other four individuals did not have significant differences in their Bray-Curtis dissimilarity among sampling sites (P-values > 0.35).

### Micro-heterogeneity among biopsy duplicates of some individuals

We further evaluated if heterogeneity existed in biopsy duplicates obtained at the same colonic location but spaced 1 cm apart. Given that the five studied individuals exhibited an average of 0.39 Bray-Curtis dissimilarity along the colon, we used the 0.39 index as a benchmark to elucidate the differences among duplicate biopsies. The data indicated that the Bray-Curtis dissimilarity index exhibited between biopsy duplicates at the same colonic location was lower than 0.39 for most individuals (Figure 3B), indicating that heterogeneity in mucosal-associated microbiota at small biogeographical distances was lower than that in the longitudinal direction. However, there were three exceptions, namely, between right colon duplicates in individual A (Bray-Curtis dissimilarity index  =  0.40), between rectal duplicates in individual C (Bray-Curtis dissimilarity index  =  0.43), and between left colon duplicates in individual D (Bray-Curtis dissimilarity index  =  0.58). In addition, the biopsy duplicates retrieved from the right colon of individual A and the left colon of individual D exhibited a 1.6-fold and 2.3-fold difference in the microbial richness (defined as number of OTUs at 97% sequence similarity), respectively (Figure S1B).

### Correlation between acidomucin staining and the microbial community

We attempted to determine if micro-heterogeneity in the localized mucosal-associated microbiota would correlate to localized biochemical differences. Based on the acidomucin staining, interindividual differences in the abundance of sialomucins and sulfomucins were apparent (Table 1). Unlike the microbial community, which exhibited no observable clustering differences in relation to the colonic site (Figure 2), the abundance of acidomucins (i.e., combination of both sialo- and sulfomucins) in the rectum generally clustered together and apart from those in the left and right colon (Figure S3). We further examined the correlation between the abundance of acidomucins goblet cells and the microbial community, and observed a weak but significant correlation between the two parameters (Mantel correlation  =  0.13, P-value  =  0.04).

## Discussion

The mucosa is the anatomical site at which the host first encounters gut microorganisms [16], and it plays a key role in intestinal homeostasis [17], [18], [19]. We inferred that microorganisms that are associated with the colonic mucosa would play a significant role in the modulation of host health, and are worthy of a closer examination. To date, our understanding of mucosal-associated microbiota has been derived primarily from culture-based analysis as well as from earlier studies utilizing microbial profiling methods (e.g., DGGE and T-RFLP) [20]. A limited number of deep sequencing studies have been conducted to elucidate the phylogenetic identities of colonic mucosal-associated microbes, in particular, the microbiota from healthy individuals (Table 3). This study therefore utilized 16S rRNA-based pyrosequencing technology to provide a comprehensive insight to the baseline microbial community that is closely associated with the colonic mucosa.

**Table 3:** List of selected publications that utilized sequencing to examine mucosal-associated microbiota in healthy individuals.

Intestinal site	Sample description	Approach	Key findings	Ref.
**Healthy state**				
Right colon, left colon, rectum, and pooled stool samples	Biopsies from five healthy individuals at the specified colonic site, duplicate at each site for each individual, five pooled stool samples from 20 other individuals	16S rRNA-based pyrosequencing ; total of 165,953 16S rRNA sequences	Predominant mucosal-associated phyla include Firmicutes (50.9%), Bacteroidetes (40.2%) and Proteobacteria (8.6%); *Bacteroides* and LAB were ubiquitously present in > 90% of studied biopsy samples, and were in high abundances; average 61% Bray-Curtis similarity along the colon; micro-heterogeneity observed in biopsies duplicates of close proximities for three individuals	This study
Rectum, stool samples	Biopsies and stool samples from nine healthy individuals, four replicates for each individual and pooled for molecular analysis. No bowel preparation was done prior colonoscopy	16S rRNA clone libraries and sequencing; total of 13,368 partial length (700 nt) 16S rRNA sequences	Predominant phyla in both rectal mucosa and stools include Firmicutes (59.4%) and Bacteroidetes (36.1%); core family in the rectal biopsy samples include unclassified Actinobacteria, Ruminococcaceae, Porphyromonadaceae, Lachnospiraceae, Bacteroidaceae	[34]
Pooled mucosal samples from cecum, right colon, transverse colon, left colon, sigmoid colon, rectum; stool samples	Biopsies from three healthy individuals, no sample duplicates collected	16S rRNA clone libraries and sequencing; total of 11,831 bacterial and 1,524 archaeal full length 16S rRNA sequences	Predominant phyla in both mucosa and stool include Firmicutes (76.2%) and Bacteroidetes (16.5%); interindividual differences account for the greatest amount of variability; relative lack of variation among mucosa sites, although some degree of micro-heterogeneity observed in some individuals	[35]
Mucosal samples from jejunum, distal ileum, right colon and rectum	Biopsies from one healthy individual, duplicates collected at each location	16S rRNA clone libraries and sequencing; total of 347 partial length (850bp) 16S rRNA sequences	Predominant phyla at the colonic sites include Firmicutes (53%) and Bacteroidetes (35.5%); insignificant differences in bacterial diversity among samples retrieved from distal ileum, ascending colon and rectum ; significant difference was observed when compared against the jejunum bacterial diversity	[36]

It is interesting to note that two of the five individuals in this study were spousal partners living in the same household. Yet, the mucosal-associated microbiota in both individuals clustered apart on the MDS, indicating that each host harbors a unique mucosal-associated microbiota and that the host effect is a significant confounder on the gut microbiota. Our data demonstrate that despite interindividual differences, the five individuals harbored consistently high predominance of Firmicutes (50.9%) and Bacteroidetes (40.2%), and the abundances of both phyla closely approximate findings from previous studies (Table 3 and Table 4). Closer examination at a finer taxonomical resolution however revealed slight differences in the abundance of bacterial groups that were ubiquitously observed in the mucosa. To illustrate, this study showed a high abundance of *Bacteroides* spp. and lactic-acid bacteria (LAB) including *Leuconostoc* spp., *Weissella* spp. and *Lactococcus* spp. among the five individuals. In contrast, previous studies reported *Bacteroides* spp. and microbial groups belonging to Ruminococcaceae and Lachnospiraceae to be more commonly found in mucosa-associated microbiota (Table 3 and Table 4). Besides interindividual differences, the variations in our findings may also be explained by slight differences in experimental technicalities such as sequencing depth and DNA extraction protocols.

**Table 4:** List of selected publications that utilized sequencing to examine mucosal-associated microbiota in diseased individuals.

Intestinal site	Sample description	Approach	Key findings	Ref.
**Diseased state**				
Inflamed and non-inflamed colonic sites of same individual	Six patients with Crohn's disease, six patients with ulcerative colitis, five healthy individuals	16S rRNA-based clone libraries and sequencing ; total of 10,010 full length 16S rRNA sequences	Predominant mucosal-associated phyla include Firmicutes (51.8%), Bacteroidetes (41.1%) and Proteobacteria (∼6%); differences in microbial community between inflamed and non-inflamed biopsies but no specific bacterial species were consistently associated with the inflamed biopsies	[37]
Stool samples, for the entire cohort. Ileum and colon for a subset of individuals	Stools were collected from total of 40 twin pairs, discordant or concordant for ulcerative colitis and Crohn's disease. Mucosal biopsies were analyzed from the cohort subset (n = 9 twin pairs)	16S rRNA-based pyrosequencing; total of 248,320 16S rRNA sequences	Predominant mucosal-associated bacterial families include Lachnospiraceae, Bacteroidaceae and Ruminococcaceae; defined healthy stool microbiome as bacterial OTUs that were present in > 50% of healthy subjects; total of 87 OTUs in the core microbiome, predominantly represented by Firmicutes and Bacteroidetes, particularly Lachnospiraceae, Ruminococcaceae, and *Bacteroides*	[38]
Normal rectum mucosa	Biopsies from 21 individuals with adenoma, and 23 non-adenoma controls	16S rRNA clone libraries and sequencing, Fluorescent in-situ hybridization (FISH), Terminal restriction fragment length polymorphism (T-RFLP); total of 335 clones were sequenced	Predominant phyla include Firmicutes (62%), Bacteroidetes (26%) and Proteobacteria (11%); *Bacteroides* spp. were the most predominant genus in the mucosal-associated microbiota of healthy controls, followed by *Subdoligranulum*, *Faecalibacterium*, *Coprococcus*, *Ruminococcus*, *Dorea*, *Roseburia*; biopsies obtained from individuals with adenoma are associated with a higher proportions of *Faecalibacterium* and *Dorea*, and a lower proportions of *Bacteroides* and *Coprococcus*	[11]

Regardless, because of their high abundance and persistence in the five individuals, *Bacteroides* spp. and LAB are postulated to form the keystone groups in the breakdown of carbohydrates to provide to the metabolic needs of these five individuals. In the human intestinal tract, *Bacteroides* spp. and related species hydrolyze complex dietary polysaccharides [21], [22], [23], and can also degrade endogenous secretions such as exfoliated epithelial cells and mucus fragments as well as residual dietary polysaccharides in the distal colon and rectum [24]. Their versatility in utilizing a broad array of polysaccharides may account for their predominance, and possibly, their role in sustaining the presence of other microbes in the intestinal tract. In contrast, *Leuconostoc*, *Weissella* and *Lactococcus*, primarily utilize readily fermentable sugars (e.g., hexoses and pentoses) to produce lactic acid [25]. Given the relatively low abundance of such simple carbohydrates in the distal colon and rectum, the provision of substrates for LAB may depend on the degradation of complex polysaccharides by *Bacteroides* spp. The utilization of different substrates and the resulting micro-niches may explain the inverse relationship in the abundance of *Bacteroides* and LAB that was observed in the biopsy samples in close proximity to each other (Figure S4).

Our findings further indicate that some individuals exhibited a certain extent of micro-heterogeneity, both along the colon (i.e., 45% similarity for individual E) and in between locations that were 1 cm apart (i.e., on average 53% similarity for biopsy duplicates that differed in individual A, C and D). Numerous factors possibly account for the presence of micro-heterogeneity in the mucosal-associated microbiota. For example, diverticulosis in the right colon of individual E (Table 1) may have perturbed the localized microbial populations, and resulted in the relatively higher micro-heterogeneity along the colon for this individual. Alternatively, host genetics and dietary preferences are also major factors influencing the differentiation of gut microbiota [26], [27]. In addition, mucins (i.e., neutral mucins and acidomucins) are a source of endogenous substrates for the mucosal-associated bacterial fermentation, and interindividual differences in the mucin content may therefore be a factor to explain for differences in gut microbiota [28]. Among the mucins, acidomucins are the predominant mucin type in the colon [29]. The presence of sulfate and sialic acids on the carbohydrate chains of acidomucins result in higher viscosity and acidity that may be resistant to mucosal-associated bacterial fermentation [30]. We inferred that varying concentration of acidomucins in an individual can in turn lead to differences in the bioavailability of endogenous substrates, which may correlate to the micro-heterogeneity of colonic mucosal-associated microbiota.

To address this inference, we observed localized differences in the abundance of acidomucin-positive goblet cells, along and within the colon of an individual. We further found that the abundance of acidomucin in an individual is weakly correlated with the mucosal-associated microbiota (Mantel correlation  =  0.13, P-value  =  0.04). Although weak, this correlation indicates that the micro-heterogeneity in mucosal-associated microbiota may relate, in part, to localized variation in the biochemical environment associated with the duplicate biopsies. This observation is in agreement with published findings, which reported that resident mucolytic bacteria may differ among individuals according to the specific carbohydrate composition of intestinal mucins [31], [32]. In this study, the weak correlation and relatively small sample size did not permit a conclusive identification of the bacterial groups that were associated with variations in the acidomucins abundance. However, past studies have found that sulfate-reducing bacteria (e.g., *Desulfotomaculum* spp. and *Desulfobacter* spp.) were more abundant in gastrointestinal sites with greater numbers of sulfomucin-containing goblet cells [12], [13]. In addition, *Bacteroides fragilis* which can cleave sulfate from sulfomucin and in turn utilize the remaining desulfated mucins as carbon and energy sources, is also likely to correlate to the abundance of acidomucins [33]. Future studies involving a larger sample size would have to be conducted to verify the occurrence of micro-heterogeneity in colonic mucosa, as well as the potential correlation of microbial micro-heterogeneity to localized biochemical characteristics.

In recent years, comparative studies which aim to evaluate for the role of gut microbiota in relation to gastrointestinal diseases are increasingly common. One of the common approaches to addressing this aim is to examine differences in the gut microbiota of diseased and adjacent healthy mucosal samples. To access the mucosal samples, it is necessary to perform bowel cleansing prior colonoscopy, particularly when sampling the right and transverse colon. The bowel cleansing preparation may have altered the loosely adherent microbiota and the lumen microbiota, and in turn led to underestimation of the diversity or abundance of the examined microbial communities. However, standardized cleansing methodologies were used for collection of all biopsy samples, hence validating the relative differences observed in this study. Our findings revealed that certain individuals have micro-heterogeneity in their gut microbiota at localized colonic sites, and this micro-heterogeneity may potentially confound subsequent comparative analysis. To overcome these issues, it is likely that multiple biopsy duplicate pairs would have to be sampled at each particular colonic site, alongside the stool samples from the same individual, to better evaluate the extent to which dysbiosis affects host health status.

In summary, our examination with 16S rRNA-based pyrosequencing provided insight into the mucosal-associated microbiota in five healthy individuals. The data further emphasize that each individual is unique in their abundance of particular microbial taxa, and that micro-heterogeneity is in part due to localized biochemical differences, which may exist in some host individuals.

## Supporting Information

Figure S1 **Rarefaction curves and microbial richness.** (A) Rarefaction curves of mucosal-associated microbiota obtained from left colon (LC), right colon (RC) and rectum (RE) of Individual A, Individual B, Individual C, Individual D, and Individual E. At each sampling site, two biopsy samples were retrieved and denoted as 1 and 2, respectively. Rarefaction curves of stool microbiota from pooled stools were also shown. (B) Microbial richness of mucosal-associated microbiota in individuals A to E, and in the stool microbiota. Microbial richness was defined as the number of operational taxonomic units (OTUs) identified at 97% 16S rRNA gene similarity, and based upon 4000 pyrotags.
(PDF)

Figure S2 **Heat map illustrating the presence of predominant bacterial groups with relative abundance >1% of total microbial community.** Columns 1 and 2 denote the microbiota present in both left colon biopsy duplicates. Columns 3 and 4 denote the microbiota present in both right colon biopsy duplicates. Columns 5 and 6 denote the microbiota present in both rectum biopsy duplicates. Columns 7 to 11 denote the stool microbiota.
(PDF)

Figure S3 **Multidimensional scaling plot (MDS) of abundance of acidomucins (i.e. combination of sialo- and sulfomucins) in the biopsy samples of individuals A to E.** Compared to the left and right colon biopsies, the abundance of acidomucins in rectal biopsies were generally more similar and clustered closer in the MDS (shown within the dotted oval).
(TIF)

Figure S4 **Heat plot illustrating the abundance difference in the bacterial groups of biopsy duplicates in Individual A (A.Biopsy-1 and A.Biopsy-2),** Individual C (C.Biopsy-1 and C.Biopsy-2) and Individual D (D.Biopsy-1 and D.Biopsy-2).
(TIF)

Table S1 **Number of 16S pyrotags obtained for each sample.** Abbreviations RC, LC, and RE denote right colon, left colon and rectum, respectively.
(DOC)

Table S2 **Regression analyses of rarefaction curves.** Double hyperbola curve model was chosen to describe the trajectory of the rarefaction curves. The number of OTUs (97% similarity) was estimated based on 4000 pyrotag reads. Abbreviations RC, LC, and RE denote right colon, left colon and rectum, respectively.
(DOC)

Material S1 **Consensus model OTU sequences for ubiquitous bacterial groups present in the mucosa of the five individuals.**
(XLSX)
