# Supplementary figures and images for: Pyrosequencing-Based Analysis of the Mucosal Microbiota in Healthy Individuals Reveals Ubiquitous Bacterial Groups and Micro-Heterogeneity
Source: PLoS One. 2011 Sep 22;6(9):e25042. doi: 10.1371/journal.pone.0025042 (PMC3178588; doi:10.1371/journal.pone.0025042)

Figure S1A

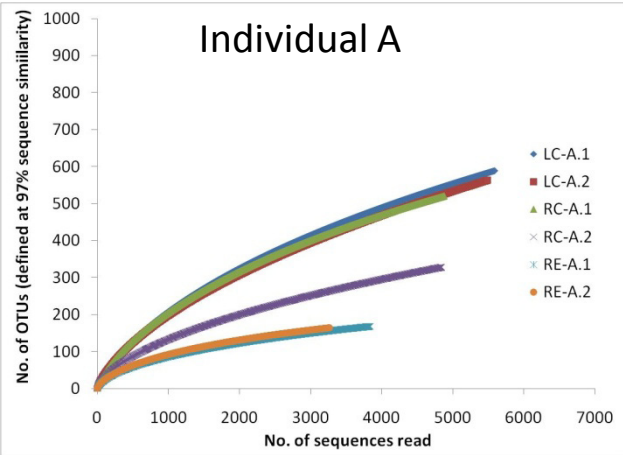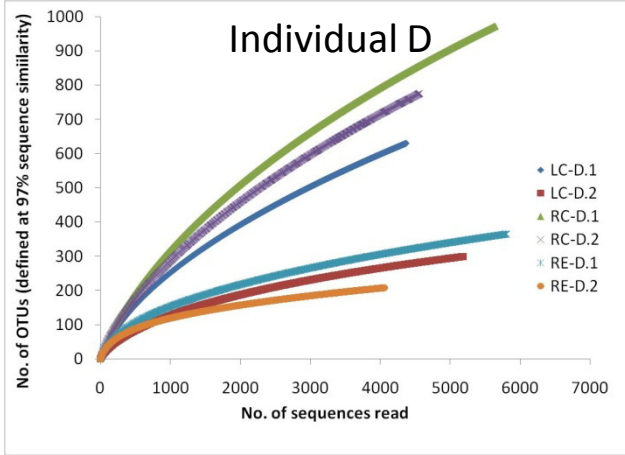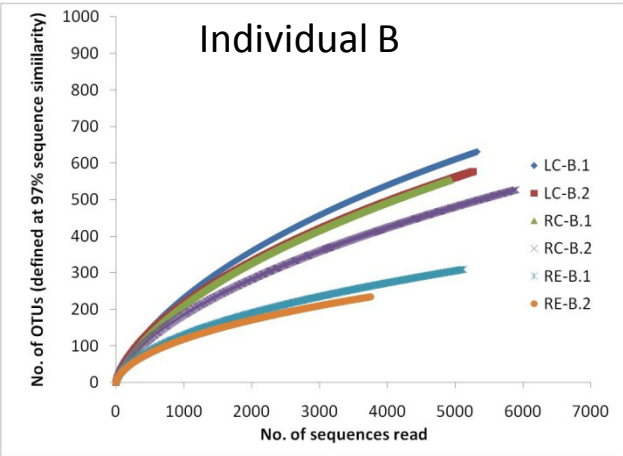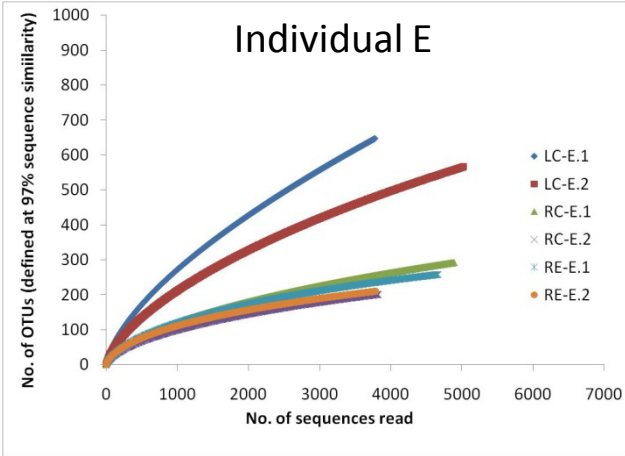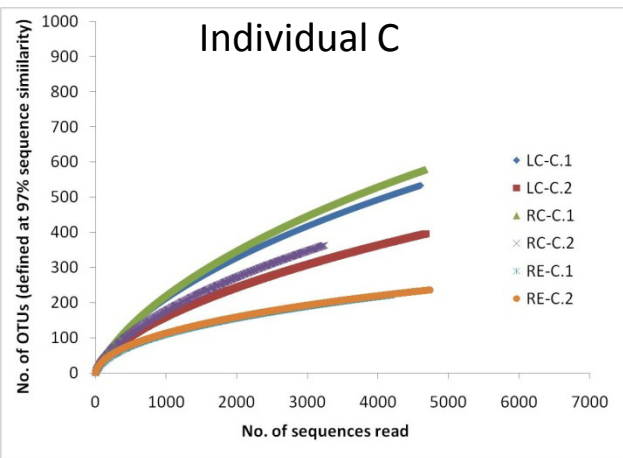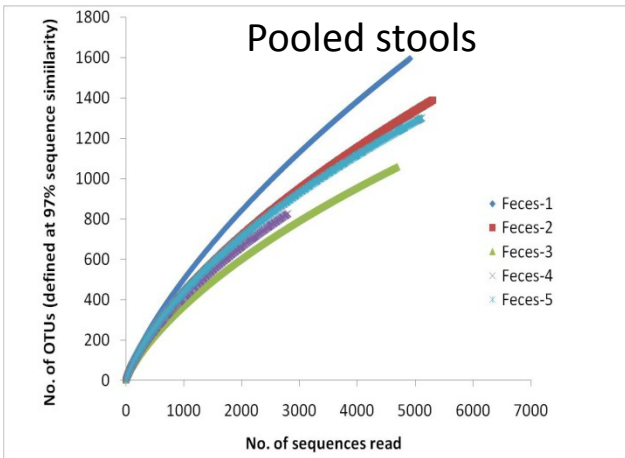

**Figure S1B**

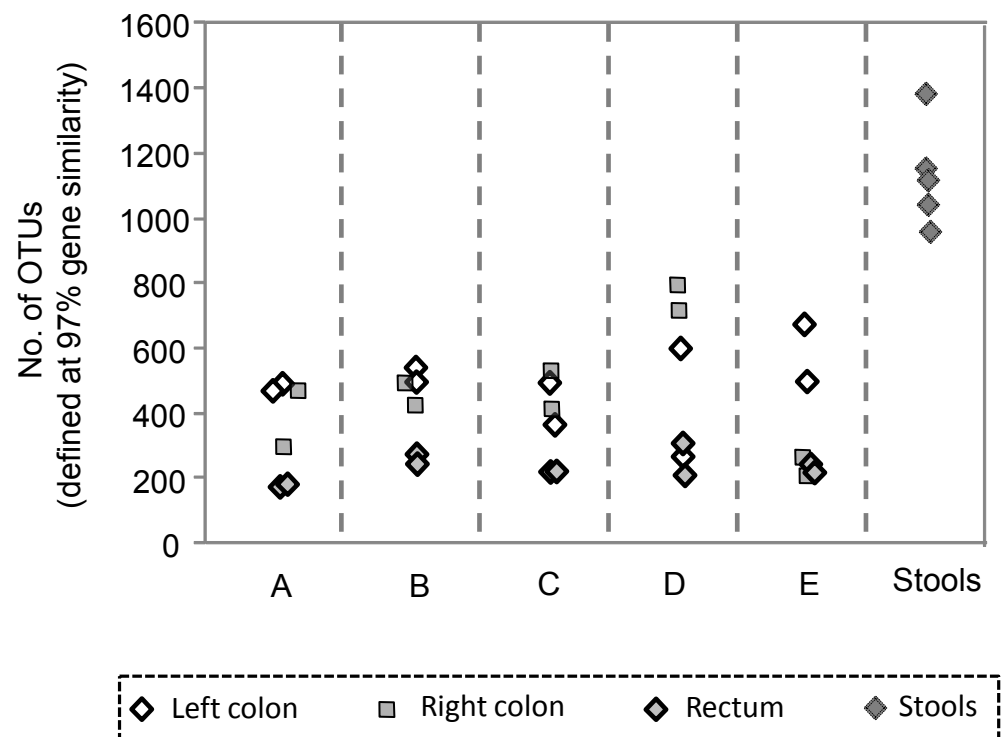

Supplement: Figure S1 — Rarefaction curves and microbial richness. (A) Rarefaction curves of mucosal-associated microbiota obtained from left colon (LC), right colon (RC) and rectum (RE) of Individual A, Individual B, Individual C, Individual D, and Individual E. At each sampling site, two biopsy samples were retrieved and denoted as 1 and 2, respectively. Rarefaction curves of stool microbiota from pooled stools were also shown. (B) Microbial richness of mucosal-associated microbiota in individuals A to E, and in the stool microbiota. Microbial richness was defined as the number of operational taxonomic units (OTUs) identified at 97% 16S rRNA gene similarity, and based upon 4000 pyrotags. (PDF) [file pone.0025042.s001.pdf]

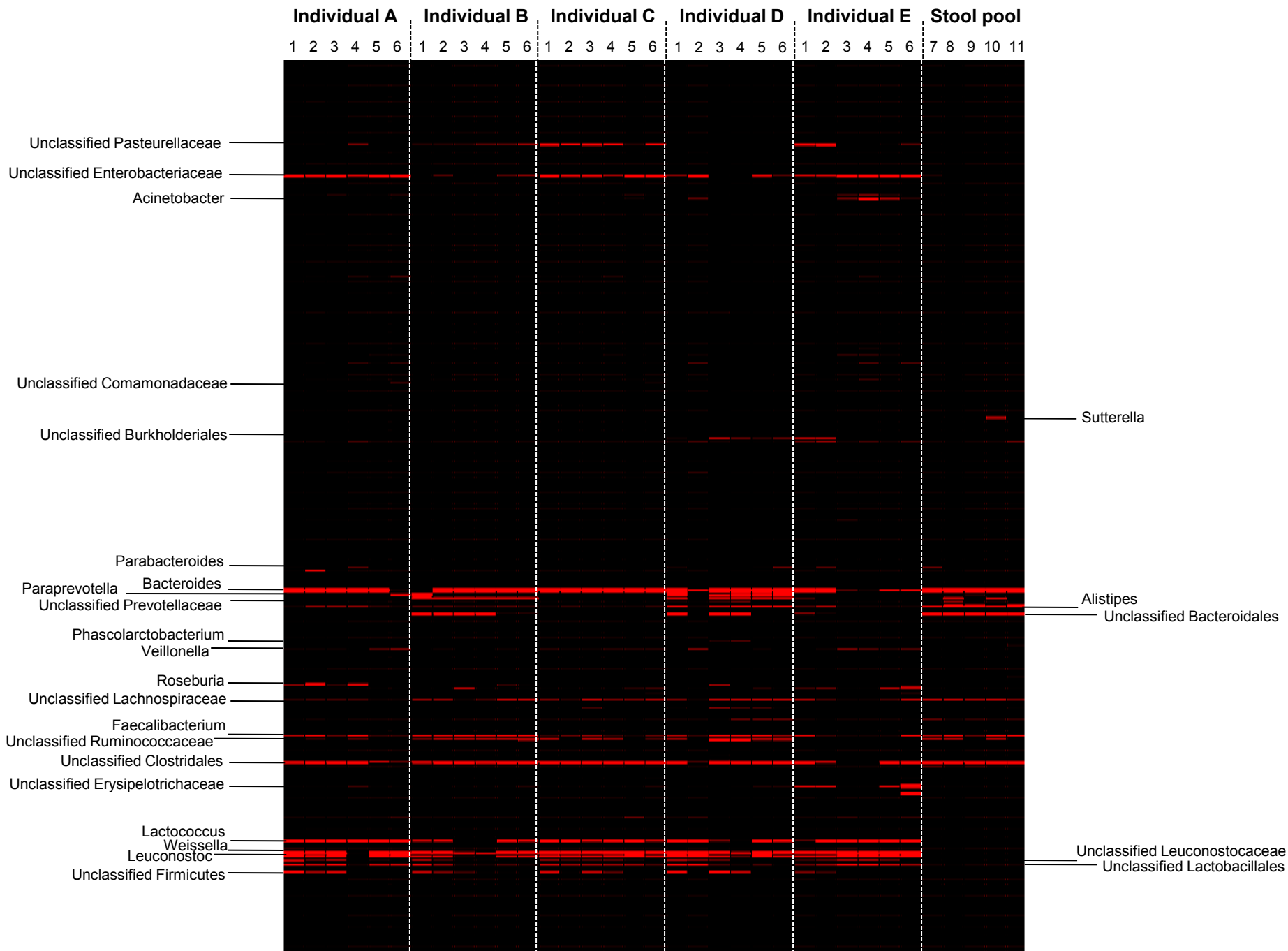

Supplement: Figure S2 — Heat map illustrating the presence of predominant bacterial groups with relative abundance >1% of total microbial community. Columns 1 and 2 denote the microbiota present in both left colon biopsy duplicates. Columns 3 and 4 denote the microbiota present in both right colon biopsy duplicates. Columns 5 and 6 denote the microbiota present in both rectum biopsy duplicates. Columns 7 to 11 denote the stool microbiota. (PDF) [file pone.0025042.s002.pdf]

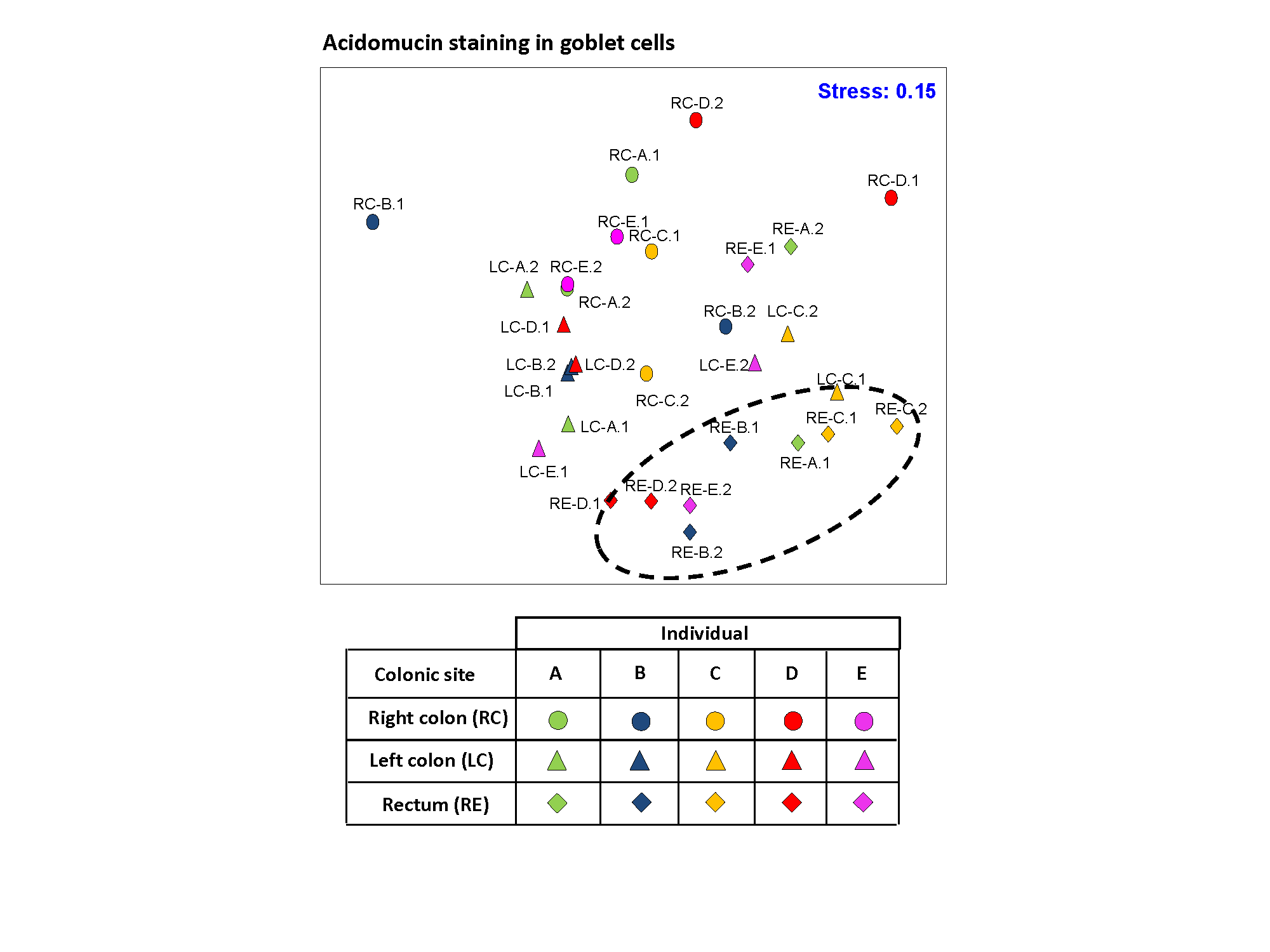

Supplement: Figure S3 — Multidimensional scaling plot (MDS) of abundance of acidomucins (i.e. combination of sialo- and sulfomucins) in the biopsy samples of individuals A to E. Compared to the left and right colon biopsies, the abundance of acidomucins in rectal biopsies were generally more similar and clustered closer in the MDS (shown within the dotted oval). (TIF) [file pone.0025042.s003.tif]

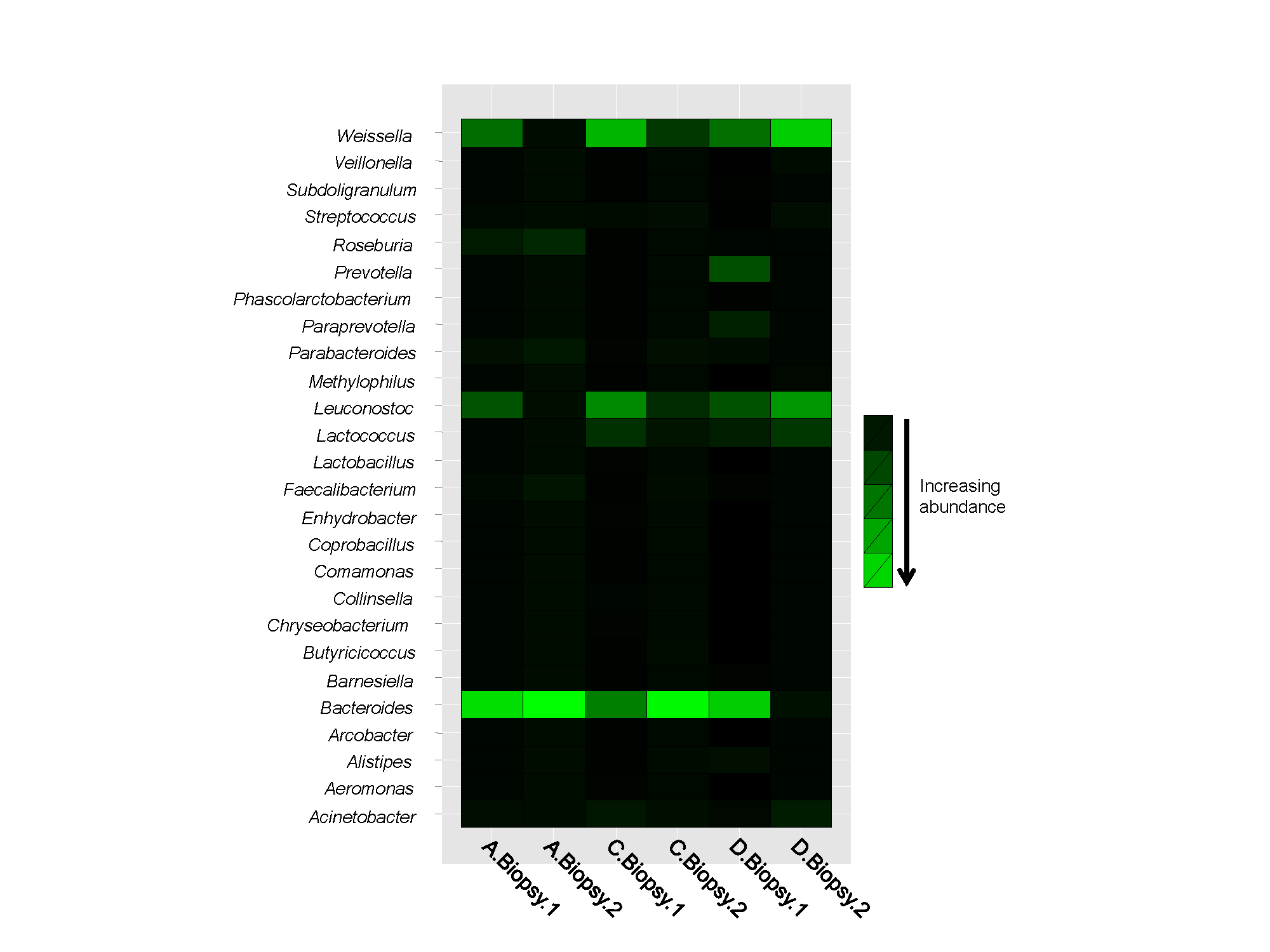

Supplement: Figure S4 — Heat plot illustrating the abundance difference in the bacterial groups of biopsy duplicates in Individual A (A.Biopsy-1 and A.Biopsy-2), Individual C (C.Biopsy-1 and C.Biopsy-2) and Individual D (D.Biopsy-1 and D.Biopsy-2). (TIF) [file pone.0025042.s004.tif]
