# Supplementary material for: Pyrosequencing-Based Analysis of the Mucosal Microbiota in Healthy Individuals Reveals Ubiquitous Bacterial Groups and Micro-Heterogeneity
Source: PLoS One. 2011 Sep 22;6(9):e25042. doi: 10.1371/journal.pone.0025042 (PMC3178588; doi:10.1371/journal.pone.0025042)
Supplement: Table S1 — Number of 16S pyrotags obtained for each sample. Abbreviations RC, LC, and RE denote right colon, left colon and rectum, respectively. (DOC) [file pone.0025042.s005.doc]

**Table S1.** Number of 16S pyrotags obtained for each sample. Abbreviations RC, LC, and RE denote right colon, left colon and rectum, respectively.

| **Sample name** | **Individual** | **Colonic site** | **Duplicate number** | **No. of raw pyrotags** | **No. of pyrotags after quality control** | **% of raw pyrotags removed** | **Pyrotag length (nt)** |
| --- | --- | --- | --- | --- | --- | --- | --- |
| RC-A.1 | A | RC | 1 | 4970 | 4883 | 1.75 | 370 |
| RC-A.2 | A | RC | 2 | 4947 | 4845 | 2.06 | 368 |
| LC-A.1 | A | LC | 1 | 5677 | 5593 | 1.48 | 370 |
| LC-A.2 | A | LC | 2 | 5590 | 5491 | 1.77 | 370 |
| RE-A.1 | A | RE | 1 | 3901 | 3843 | 1.49 | 370 |
| RE-A.2 | A | RE | 2 | 3315 | 3267 | 1.45 | 371 |
| RC-B.1 | B | RC | 1 | 5008 | 4927 | 1.62 | 368 |
| RC-B.2 | B | RC | 2 | 6025 | 5899 | 2.09 | 368 |
| LC-B.1 | B | LC | 1 | 5402 | 5327 | 1.39 | 369 |
| LC-B.2 | B | LC | 2 | 5424 | 5269 | 2.86 | 369 |
| RE-B.1 | B | RE | 1 | 5231 | 5124 | 2.05 | 369 |
| RE-B.2 | B | RE | 2 | 3820 | 3758 | 1.62 | 369 |
| RC-C.1 | C | RC | 1 | 4773 | 4672 | 2.12 | 369 |
| RC-C.2 | C | RC | 2 | 3299 | 3244 | 1.67 | 369 |
| LC-C.1 | C | LC | 1 | 4693 | 4609 | 1.79 | 369 |
| LC-C.2 | C | LC | 2 | 4753 | 4681 | 1.51 | 370 |
| RE-C.1 | C | RE | 1 | 4231 | 4188 | 1.02 | 370 |
| RE-C.2 | C | RE | 2 | 4827 | 4742 | 1.76 | 369 |
| RC-D.1 | D | RC | 1 | 5729 | 5601 | 2.23 | 369 |
| RC-D.2 | D | RC | 2 | 4647 | 4569 | 1.68 | 369 |
| LC-D.1 | D | LC | 1 | 4427 | 4368 | 1.33 | 369 |
| LC-D.2 | D | LC | 2 | 5264 | 5186 | 1.48 | 371 |
| RE-D.1 | D | RE | 1 | 5943 | 5811 | 2.22 | 370 |
| RE-D.2 | D | RE | 2 | 4156 | 4062 | 2.26 | 369 |
| RC-E.1 | E | RC | 1 | 4997 | 4892 | 2.10 | 371 |
| RC-E.2 | E | RC | 2 | 3883 | 3829 | 1.39 | 371 |
| LC-E.1 | E | LC | 1 | 3859 | 3782 | 2.00 | 369 |
| LC-E.2 | E | LC | 2 | 5121 | 5023 | 1.91 | 369 |
| RE-E.1 | E | RE | 1 | 4751 | 4666 | 1.79 | 371 |
| RE-E.2 | E | RE | 2 | 3850 | 3791 | 1.53 | 370 |
| Stools-1 | Pooled from a total of 20 individuals | Stools | 1 | 5130 | 4910 | 4.29 | 368 |
| Stools-2 |  | Stools | 2 | 5385 | 5306 | 1.47 | 368 |
| Stools-3 |  | Stools | 3 | 4814 | 4686 | 2.66 | 368 |
| Stools-4 |  | Stools | 4 | 2869 | 2806 | 2.20 | 368 |
| Stools-5 |  | Stools | 5 | 5242 | 5123 | 2.27 | 368 |
