# Supplementary material for: Pyrosequencing-Based Analysis of the Mucosal Microbiota in Healthy Individuals Reveals Ubiquitous Bacterial Groups and Micro-Heterogeneity
Source: PLoS One. 2011 Sep 22;6(9):e25042. doi: 10.1371/journal.pone.0025042 (PMC3178588; doi:10.1371/journal.pone.0025042)
Supplement: Table S2 — Regression analyses of rarefaction curves. Double hyperbola curve model was chosen to describe the trajectory of the rarefaction curves. The number of OTUs (97% similarity) was estimated based on 4000 pyrotag reads. Abbreviations RC, LC, and RE denote right colon, left colon and rectum, respectively. (DOC) [file pone.0025042.s006.doc]

**Table S2.** Regression analyses of rarefaction curves. Double hyperbola curve model was chosen to describe the trajectory of the rarefaction curves. The number of OTUs (97% similarity) was estimated based on 4000 pyrotag reads. Abbreviations RC, LC, and RE denote right colon, left colon and rectum, respectively.

| Double rectangular hyperbola curve is described by the following algorithm:  y = ax/(b+x) + cx/(d+x) + ex  where y = number of OTUs defined at 97% gene similarity  x = number of 16S pyrotags sequenced  a, b, c, d and e = numerical constants | | | | | | | | | | | | | | | | | |
| --- | --- | --- | --- | --- | --- | --- | --- | --- | --- | --- | --- | --- | --- | --- | --- | --- | --- |
| Sample name | Individual | | Colonic site | | Duplicate number | | a | b | | | c | d | | e | | Value of y (i.e., no. of OTU) when x = 4000 | |
| RC-A.1 | A | | RC | | 1 | | 553.3681 | 3386.119 | | | 60.5928 | 197.8838 | | 0.02772 | | 468 | |
| RC-A.2 | A | | RC | | 2 | | 252.2208 | 2666.945 | | | 42.80165 | 121.8788 | | 0.02553 | | 295 | |
| LC-A.1 | A | | LC | | 1 | | 528.3728 | 3136.435 | | | 50.75378 | 165.5913 | | 0.03603 | | 489 | |
| LC-A.2 | A | | LC | | 2 | | 395.1874 | 2649.294 | | | 52.40143 | 173.2098 | | 0.04483 | | 467 | |
| RE-A.1 | A | | RE | | 1 | | 25.70443 | 75.28519 | | | 141.6893 | 1860.632 | | 0.01236 | | 171 | |
| RE-A.2 | A | | RE | | 2 | | 21.03893 | 60.32128 | | | 150.2606 | 1525.003 | | 0.01257 | | 180 | |
| RC-B.1 | B | | RC | | 1 | | 429.4904 | 2555.548 | | | 52.7027 | 128.8524 | | 0.0447 | | 492 | |
| RC-B.2 | B | | RC | | 2 | | 394.494 | 3029.407 | | | 58.04567 | 137.3003 | | 0.0356 | | 423 | |
| LC-B.1 | B | | LC | | 1 | | 620.277 | 3775.483 | | | 81.35943 | 228.0842 | | 0.03578 | | 539 | |
| LC-B.2 | B | | LC | | 2 | | 509.9639 | 3416.329 | | | 71.58414 | 180.8544 | | 0.03775 | | 495 | |
| RE-B.1 | B | | RE | | 1 | | 33.83449 | 63.82592 | | | 216.3624 | 1904.355 | | 0.02307 | | 272 | |
| RE-B.2 | B | | RE | | 2 | | 29.09595 | 56.96628 | | | 175.0383 | 1537.536 | | 0.02169 | | 242 | |
| RC-C.1 | C | | RC | | 1 | | 641.9215 | 4044.729 | | | 71.24333 | 215.0698 | | 0.03559 | | 529 | |
| RC-C.2 | C | | RC | | 2 | | 286.6797 | 1980.722 | | | 40.63163 | 118.836 | | 0.04505 | | 411 | |
| LC-C.1 | C | | LC | | 1 | | 491.1654 | 3045.626 | | | 56.77457 | 154.9224 | | 0.03964 | | 492 | |
| LC-C.2 | C | | LC | | 2 | | 311.3455 | 2785.995 | | | 51.49162 | 131.7017 | | 0.0323 | | 363 | |
| RE-C.1 | C | | RE | | 1 | | 46.54365 | 125.5411 | | | 165.0879 | 2095.43 | | 0.016 | | 217 | |
| RE-C.2 | C | | RE | | 2 | | 49.21229 | 100.5516 | | | 211.2616 | 2719.239 | | 0.01135 | | 219 | |
| RC-D.1 | D | | RC | | 1 | | 922.2184 | 3934.912 | | | 87.84934 | 235.6 | | 0.06136 | | 793 | |
| RC-D.2 | D | | RC | | 2 | | 683.3745 | 3219.022 | | | 64.31969 | 147.8601 | | 0.06858 | | 715 | |
| Double rectangular hyperbola curve is described by the following algorithm:  y = ax/(b+x) + cx/(d+x) + ex  where y = number of OTUs defined at 97% gene similarity  x = number of 16S pyrotags sequenced  a, b, c, d and e = numerical constants | | | | | | | | | | | | | | | | | |
| Sample name | | Individual | | Location | | Duplicate number | a | | b | c | | | d | | e | | Value of y when x = 4000 |
| LC-D.1 | | D | | LC | | 1 | 457.7048 | | 2383.202 | 62.93558 | | | 165.1764 | | 0.06267 | | 598 |
| LC-D.2 | | D | | LC | | 2 | 279.3611 | | 2650.755 | 41.66925 | | | 143.8145 | | 0.01424 | | 265 |
| RE-D.1 | | D | | RE | | 1 | 81.02798 | | 172.9768 | 271.5529 | | | 3186.137 | | 0.01914 | | 305 |
| RE-D.2 | | D | | RE | | 2 | 48.85206 | | 87.15858 | 117.3157 | | | 1030.818 | | 0.01648 | | 207 |
| RC-E.1 | | E | | RC | | 1 | 235.8549 | | 2502.76 | 36.41085 | | | 116.711 | | 0.02059 | | 263 |
| RC-E.2 | | E | | RC | | 2 | 187.9019 | | 2404.713 | 33.65558 | | | 104.0631 | | 0.01386 | | 206 |
| LC-E.1 | | E | | LC | | 1 | 367.9551 | | 2050.245 | 68.1024 | | | 130.9913 | | 0.09073 | | 672 |
| LC-E.2 | | E | | LC | | 2 | 487.4886 | | 3210.018 | 60.3032 | | | 120.7454 | | 0.04184 | | 496 |
| RE-E.1 | | E | | RE | | 1 | 41.68913 | | 131.8376 | 275.0175 | | | 2788.176 | | 0.00976 | | 241 |
| RE-E.2 | | E | | RE | | 2 | 28.0008 | | 62.76019 | 162.4633 | | | 1388.274 | | 0.01683 | | 215 |
| Stools-1 | | Pooled from a total of 20 individuals | | Stools | | 1 | 1612.555 | | 5160.275 | 126.8368 | | | 290.736 | | 0.13998 | | 1382 |
| Stools-2 | |  |  | Stools | | 2 | 1151.15 | | 4114.674 | 117.0817 | | | 290.1641 | | 0.11898 | | 1153 |
| Stools-3 | |  |  | Stools | | 3 | 966.426 | | 3734.837 | 91.73993 | | | 217.4267 | | 0.09259 | | 957 |
| Stools-4 | |  |  | Stools | | 4 | 787.3581 | | 2569.518 | 84.69506 | | | 193.1905 | | 0.1201 | | 1041 |
| Stools-5 | |  |  | Stools | | 5 | 1239.756 | | 4047.504 | 120.3274 | | | 255.6886 | | 0.09667 | | 1116 |
